# Bibliographical Notices

**Published:** 1843-01-01

**Authors:** 


### Periscope;

OR,

### CIRCUMSPECTIVE REVIEW.

" Ore trahit quodcunque potest, atque addit acervo."

### Notices of some New Works.

Ueber die Abhangigheit der Physischen Populationskrafte von der einfachsten Grundstoffen der Natur; mit specieller Anwendung auf die Bevolkerungs-Statistik von Belgien, von Dr. *Ferdinand Gobbi.* Liepzig and Paris.

On the Dependence of the Physical Population-powers on the Simplest Elements of Nature; with Special Application to the Population-statistics of Belgium. By Dr. *Gobbi.*

Before we had waded through the first page of the Introduction to this work, we were forcibly reminded of the cyclical poet mentioned by Horace, who commenced his poem on the Trojan war with an account of the eggs laid and hatched by mother Leda, and one of the chicks of which was the unlucky Helen, " causa teterrima belli." Dr. Gobbi commences his work, the avowed object of which is to point out the connexion between the physical population-powers of the earth and the simplest elements of Nature, by a very detailed and elaborate account of what is now called the *Nebular Theory of the Universe,* a theory which, if we mistake not, had its origin in the celebrated hypothesis of Laplace; according to which, all the planets, now constituting the Solar System, were formed out of a very thin, luminous, and widely extended atmosphere, which surrounded the body of the Sun, and which, together with him, revolved on an axis.

The connexion between such an origin of our own little planet, the earth, and its powers of maintaining and propagating human existence, does not, to say the least, appear very striking at first sight.

To those, however, who may feel disposed, and have sufficient time, to wade through this rather heavy, and certainly abstruse subject, a subject, moreover, for the due understanding of which, no small degree of imaginative exertion is required, such connexion will not appear quite so improbable. For our parts, however, we must decline attempting any thing like an analysis of this very profound work; it would, indeed, be quite out of place in the pages of a Journal, the prominent character, and legitimate object of which has ever been the discussion and elucidation of matters more immediately connected with practical medicine. We shall, in the meantime, present our readers with some of the more important and practical results deduced from the principles laid down in it.

The author divides his work into three grand Sections, the first of which treats of the *Action of the Atmospheric Water on the entire Process of Organisation.* This section of the work is subdivided into five chapters. In the first is considered—

*The Influence of the Atmospheric Water on the Assimilation of Food in the First Passages.*

Our author sets out with the hypothesis of a *unity of life* which pervades the entire universe; he says, that we can account to ourselves for the existence of the universe in no other way, than by supposing a single fundamental power, *one life*, pervading all the bodies which constitute the universe, and extended to us. All the elements of these bodies we must conceive to be formed after the same type, only that this fundamental power, this life, imparts to each different qualities, according as it presents itself in them in a simple or compound form. He next divides the bodies which present themselves on the surface of our earth into two grand classes or groups, the one containing unorganised and the other organised beings, and points out the unbroken, uninterrupted chain which connects these two classes. In illustration of this same principle he adduces the Nullipori and Lithophytes, as the connecting link between the animal and the unorganized kingdom, and also the very close affinity of the Algæ, Tremelli and Ulvæ to Zoophytes and Polypi. So striking, he says, is the homogeneïty of these two kingdoms, that Sponges and Tremelli are considered by some as plants, and by others as animals. He further says, that all the marks of Heterogeneïty between organised and unorganised substances only serve to indicate a closer concatenation and a more intimate connexion, which is not only not incompatible with the idea of a single life of the universe, but is even strengthened by it; for, he says, until the individual elements of a body have received from the universe a certain degree of antithesis, which is always the consequence of a somewhat higher state of development, such a body cannot become a participator in that principle, which should be capable of uniting its individual fractions into a self-subsisting whole, and of imparting to it a substantial form, that principle, in fact, which enables it to convert foreign substances into its own nature, and of forming them into consentient organs, co-operating for one and the same end. We shall not here enter into all the details of our author's reasoning, but shall state the conclusion to which he comes, viz. that organic beings possess the power of assimilating to themselves heterogeneous substances, and also that their existence depends on the possibility of their changing the peculiar qualities of foreign bodies, so that they may be converted into nutriment for themselves.

The principle of unity, above alluded to, in the entire kingdom of nature, establishes it accordingly as an indispensable condition, that every body in nature, according to its different position, must make use of those placed beneath it in organic dignity for its own support, just as itself is marked out for the reparation of all others which are placed above it in organic development. But all substances are capable of assimilating foreign matters only by decomposing them, and reducing them to such a state, that their original nature is no longer recognisable. The facility with which this end may be accomplished, depends entirely on the degree in which the decomposition of these respective alimentary materials takes place. It may be asked here, what means does nature employ in order to facilitate this operation? To solve this question, our author enters somewhat into detail. Man, he says, the chief object of our enquiries, can maintain his own peculiar composition and vital activity immediately only by the higher and more perfectly elaborated products of the vegetable and animal kingdoms: even the animals, which serve for his nutrition, take from the external world for the support of their existence only organised and chiefly vegetable substances. As the immediate and mediate source of the support of man, we therefore consider the vegetable kingdom more appropriately than the mineral and animal kingdom. So soon as the first molecule of the first material germ of life is formed, the external world already begins to act on it, and it evinces its self-subsistence and its inner life by its capability of withstanding such aggressions. No doubt it gives off every moment a part of itself to this aggressor, but for this it is continually attracting other portions to itself; a continued reciprocal action, a never ceasing struggle, is accordingly established between this new creation and the external world; the latter wishes to rob it of its self-subsistence and of the materials appropriated to itself, whilst the former will appropriate to itself a constantly encreasing quantum of material, it will encrease, and go on to form itself according to its own fashion. The internal vital power of this new being struggles therefore with the external world about the appropriation of its material substratum. Should this internal vital power be sufficiently strong to overcome the opposition of those substances, which would thwart its progress onwards, it gains in matter, and to this matter it annexes a higher degree of vitality; if it be able only to paralyse the above-mentioned opposition, then the material loss becomes just equal to the cotemporaneous gain, and the degree of vitality continues in the same degree; but should the vital power succumb in the struggle, its material substratum vanishes every moment, and itself is constantly diminishing more and more, and approaches to that state, in which it originally presented the first molecule of its material germ to the external world for the object of the struggle. The quality of those substances, therefore, which more especially from the external world, exercises the most immediate influence on the bodies producing themselves in this way, must regulate the course of the entire process. The facility of their appropriation, the quantity of readily assimilable materials, and the predominance among them of those which are best suited to the nature of the forthcoming corpuscle, determine as it were its whole chance of success, which therefore, in other words, will depend on the degree of solution of these substances. He next proceeds to show that this solution or decomposition of these substances, so necessary for their assimilation, is regulated chiefly by water, oxygen, hydrogen, and what are usually called the imponderables. He then observes that water is the regulator of vegetation, and thence deduces the necessary dependence of the materials of the human organism on water, in as much as the vegetable substances employed for its support depend immediately on water. After proving the indispensable necessity of the presence of moisture to vegetation generally, and to individul species of vegetables in particular, he ends this, the first, chapter by concluding that "the development of all those substances, which man derives from the external world, as well from the vegetable as from the animal kingdom, for the purpose of his nutrition, depends unconditionally on the aqueous vapour suspended in the air. The first assimilation of the food, that namely whereby it is converted into a fluid, which is then by the action of atmospheric air formed into a perfectly organic material, must consequently be regulated by the atmospheric water."

The Second Chapter treats of *the Influence of the Atmospheric Water on the Respiration.*

This chapter commences by pointing out how indispensable atmospheric air is for animals and vegetables, and also the necessity of a second great metamorphosis of the nutritious juices by the air. We have here a description of the respiratory organs of plants and animals; of the qualities of atmospheric air, and of the aqueous vapour suspended in it; of the circumstances also which modify this vapour with respect to quantity; of its influence on the inhalation of oxygen and the exhalation of carbonic acid, on the absorption of nitrogen, and on the pulmonary transpiration in the human subject; in all this we do not find any thing very different from what we meet in our ordinary works on chemistry and physiology. The winding up of this chapter is, that the whole process whereby the crude chyle is fitted by the addition of atmospheric air for the reparation of the organic material which has been consumed through the vital process, is wholly dependent on the watery vapour suspended in the atmosphere.

The *Fourth Chapter* describes the influence of the atmospheric water on animal heat. Here we have an account of the warmth brought to the earth by the sun's rays. The author shews that the organic process going on within the body is the principal source of animal heat, and that the process of respiration by itself is but a modificator of it. The watery contents of the atmospheric air, and the evaporation going on from the animal body are the mediate regulators of animal heat. The aqueous vapour suspended in the atmosphere is proved to be a modifier of the animal heat from its influence in raising and depressing the temperature of the air, which variations of temperature in the atmosphere cannot but exercise considerable influence on the heat of the organism.

The conclusion which our author arrives at from the principles and reasonings contained in the first section of this work is, that the aqueous vapour suspended in the atmosphere is the regulator of all the functions, and of all the conditions of human life; the preparation of the crude nutritious fluid, its perfect organisation by respiration, the graduation of animal heat, the production of animal electricity, and the special influence of light, are unconditionally regulated by it. The author has here shown how the action of atmospheric air on the five chief regulators of life are regulated by the quantity of water contained in it. He has not, however, at the same time analysed for us the modifications which that action undergoes by reason of the difference of quality produced on an atmosphere, in consequence of the combination which it enters into with the aqueous vapour given off from the surface of the earth under so many varying circumstances.

Though the mere consideration of the elements of water, their peculiar relation to the imponderables, that of the incalculable quantum of these bodies, as well as their extreme divisibility, place it beyond doubt, that from the connexion of the proper elements of the atmosphere with the aqueous vapour under the influence of the solar rays as many characteristically different combinations proceed, as there are circumstances modifying the peculiar manner in which the aqueous vapour is developed, though also every one of the above-mentioned modified constitutions of the atmosphere divide and metamorphose the sun's rays in a peculiar manner, and consequently must impart a peculiar direction to all organic bodies in general and to the human organism in particular, as well mediately as immediately, still in this first section of the work we have no analysis of the effect of either of these many atmospheric variations on the process of digestion, nor on that of the respiration, nor on any of the other three great regulators of life. The author however ingenuously acknowledges that, in the present state of science, such minute analysis is beyond our reach.

We here close our notice of this very profound work. From the great importance which the author seems to attach to the *watery* vapour of the atmosphere in modifying the several vital functions, we have no doubt but that our friends, the hydropathists, will hail the book as a regular God-send.

---

### REASON AND INSTINCT COMPARED.

IN a very curious and amusing work, by Dr. J. C. Hall, entitled "Interesting Facts connected with the Animal Kingdom, &c." there is a chapter on the above subject, which is the only one we can notice in this Journal, though we can most confidently recommend the whole volume to the attention of our readers. We shall much condense our author's ideas, though adhere very close to his own language.

The grand resemblance between man and brutes is, that they are both material. Both have living organised bodies, produced by generation and supported by food. Both have senses, sensations, imagination, and memory. They both enjoy pleasure and suffer pain. Both have a natural propensity to self-preservation—both are liable to diseases—and both inevitably die.

Brutes have many advantages over man. They require no clothes or implements of defence. They bring into the world with them all that they want, except food, which instinct teaches them unerringly how to procure. As soon as their appetites are satisfied they are perfectly happy, neither reflecting on the past, nor concerning themselves for the future.

Man, on the other hand, is obliged to learn, to meditate, to invent, to labour, and, after all, has often difficulty in procuring the necessaries of life. His passions more frequently lead him astray than guide him to happiness.

But REASON is thrown into the scale, and gives man prerogatives over instinct. By this he is enabled to procure not only the necessaries, but the luxuries of life, and to multiply all his pleasures. Such pleasures result from knowledge, wisdom, religion, order, and virtue, and "endure for ever," whilst sensual gratifications unfit him for every thing great and dignified. Man is the only animal that is always advancing progressively in discoveries, and enlarging the sphere of knowledge. Whereas brutes remain stationary, and are incapable of soaring over others of the same species.

But if we come to a closer analysis or comparison of Reason and Instinct, we find great difficulty in drawing a clear distinction between the two. It is only by their great distinctive *effects* that we can discriminate the one from the other. Thus the elephant, the ant, the beaver, the bee, &c. shew combination, intellect, intelligence, communication, by some kind of language, and almost all the features of reason.

Still, however high the scale of intellect rises in the animals alluded to, they are bound down by Instinct to follow in the track of their progenitors, without any deviation, whereas Reason is eternally prompting *invention* which never appears in the brute.

Dr. Hall descants learnedly and ingeniously on the divine origin of the human mind or soul, and considers that, next to Revelation, the universal concurrence of all nations in the belief of an immortal soul, affords the strongest proofs of its truth. We cannot see any thing like proof in this general (for it is not a universal) concurrence. The belief that the sun daily travelled round the earth, was, at one time, just as general as the belief now in the future existence of the soul. There is, in fact, no proof, and scarcely a probability, of a future state of existence, except in Revelation—and with that we must be content.

We have thus given the substance of Dr. Hall's chapter; but we think he might have added several other distinctive characters between man and animals; or, in other words, between Reason and Instinct. One of the greatest distinctions, and which, as far as we know, has never been noticed, is the fact, that MAN is the only animal in existence who knows, or even thinks, that he must die. This piece of knowledge seems to be a necessary accompaniment of REASON, and no small drawback on the pleasures of that boasted gift! It is a prescience which embitters a great portion of the life of man!

Man, being a free agent, and not bound down, like brutes, by Instinct, the fear of death, and still more the fear of something after death, is one of the greatest checks on his evil propensities. He sees and knows that "the wages of sin are death," even in this world, and he is taught from infancy that, beyond the grave, there is another state of rewards and punishments. The wonder is, that, under this double system of fears and hopes, he should ever venture to commit a crime! Yet he does so every day and hour. This shews how strongly are the seeds of vice implanted in the human constitution! Yet these two checks, one obvious to our senses, the other prospective to our fears and hopes, are the only ones that hold society in any kind of order.

In respect to language, we have some doubts as to its being peculiar to man. Putting the peculiar cries of animals, by which they recognise their own species, out of the question, the division of labour among beavers, ants, bees, &c. can hardly be allotted without some communications very like language. But no animal, except man, can commit language to brass, marble, or paper. The cooking, clothing, calculating, and other characteristics of man, need not detain us. Much has been said about the "os hominis sublime," but the kangaroo stands very nearly as upright as man—and a pine-tree stands more erect than either. His physical organisation is not essentially different from that of the beasts of the field. It is by his mental powers alone that man can be distinguished from the animal kingdom around him.

One question remains. On which side does happiness predominate?—on that of man or that of beasts? Those who have lived longest in this world and seen most of human nature, will have little hesitation in casting the balance in favour of animals. The young and the sentimental will probably come to a different conclusion. As far as contentment and absence of misery are concerned, brutes have infinitely the advantage. Excepting those that have the misfortune to come under the yoke of bad and heartless wretches, brutes have little or no suffering, sorrow, or care. Food and shelter are all they want or wish, and there is every reason to believe that they enjoy both of these as much as their masters. They labour under neither gout nor dyspepsy—hypochondriasis, or monomania—chimerical hopes or superstitious terrors! They have but one object to pursue—their daily subsistence—and that object obtained, their care about the present, their regrets about the past, and their apprehension for the future, are null and void.

But then the pleasures of knowledge, of literature, arts and sciences! Are these unalloyed with misery? No, indeed. We have it, on the best authority, that KNOWLEDGE—

"Brought death into the world, and all our woe."

As far, then, as happiness is concerned, knowledge is rather a curse than a blessing. We have no reason to boast of that prerogative of man! But whether for good or for evil, the Almighty has thought fit to endow us with a power quite unknown to brutes, and therefore it is the duty of man to obey the will of Heaven.

---

### PATHOLOGICAL SEMEIOLOGY OF INFANCY. By Dr. *Vanier*.

(From the *Clinique des Hôpitaux des Enfans.*)

IN some of the former Numbers of our Journal we gave several extracts from this periodical, which is exclusively devoted to the consideration of the diseases of children. We now propose to give a succinct analysis of some papers by Dr. Vanier, which he intends to form a *General Pathology of Infancy*, and to serve as preparatory to the clinical studies of the diseases of infants, and to point out the best method of interrogating patients of this tender age.

The practitioner should first endeavour to ascertain the general constitution of his little patient.

The rickety constitution is not devoid of influence on other affections which may attack children.

The characters by which this constitution may be recognised are as follows: Children in this state have, generally speaking, black eye-brows, eyes blue, hair black, sometimes red, skin brown. Children affected with thoracic rickets have a timbre of voice entirely different from that observed in other children. This modification, more or less marked, according as the rickety constitution itself is more or less advanced, is analogous, with respect to age, to that which distinguishes the organ of adults affected with considerable deformation of the thorax.

The scrophulous constitution again presents the following characters: children so predisposed have generally red hair; the colour of their skin is a dull white, or a bluish white, and the veins are well marked and easily traced beneath the skin. They present an oily, œdematous aspect; one would almost say that they were affected with slight infiltration of the body. They are almost always ill-tempered; they cry for the slightest cause, or without any; they never smile, and they refuse all attempts at walking. They eat a great deal. They reject dainties of which other children are so fond, and greedily seize on a piece of plain bread. One character, which distinguishes scrophulous from rickety children is, that if you take them up into your arms, you feel, that when you hold them but feebly, notwithstanding the softness of their flesh, they sustain themselves of their own accord, and their muscles act; that if they catch you by the finger, you will feel that they grasp it with considerable force. Such are the children predisposed to scrophula; rickety children slip down between your hands for want of support on their legs; their arms, unable to sustain them, when they grasp your hands for support, are raised somewhat above their head on either side, whilst their body slips down to the ground. If you hold out your finger to them, they will not be able to grasp it. Such are the characters by which we shall be able to distinguish these two constitutions, in the first period of infancy, before they are as yet characterised by traits, which will soon impress them with so different a physiognomy.

These observations are of course applicable only to such children as are at least some months old.

In these remarks we shall always confine ourselves to cases of children at the breast; but among these we shall form a separate class of new-born children, that is, of children at least one month old, in whom the umbilical cicatrix is not complete, and who still present some of the appearances which attend or follow birth, such as jaundice, erythema, &c. We may now make a few observations on weakness as evinced at birth; this is manifested by an external state, which is always nearly the same with respect to its form, but of which there are different degrees: limbs slender, respiration difficult, painful cry, inability to retain drink, extreme debility, limbs emaciated, eyes hollow, and countenance cadaveric. The causes of this state of the system are very variable. It may be the effect of different organic lesions, or may exist without any species of lesion whatever. In order to treat this state of the system, we must appreciate its true cause, as the debility may be only apparent.

If there are certain functions in adults which must be interrogated, before medicinal treatment have as yet disturbed them, the same holds good still more in the case of children. Our method of proceeding in this particular consists in examining the infant, when it is quite tranquil (first examination), and also whilst it is in a disturbed state, (second period of examination). Certain functions must be examined during the calm state of the child, which, if examined during the disturbed state, would lead into error. The examination to be made whilst the child is asleep, regards the complexion, the expression and spontaneous movements of the face; the pulse, the pulsations of the heart and the number of inspirations. In the state of disturbance we shall ascertain such pain as may be occasioned by the various manipulations employed for forming a diagnosis, the state of the mouth, and of the chest, the quality of the cry occasioned by such measures.

*Examination of the Child during the Calm. Colour.*—Immediately after birth the child has a deep red colour, which diminishes from the third to the eighth day. This colour may be attributed to the influence of external agents on the tender integuments not yet habituated to their action; as the impression of the air, the friction by the hands of the accoucheur, and of others employed about the child. In fact the colour of new-born children recognises the same causes as those which occasion the first cries. If the deep red colour continues beyond eight or nine days, or if after the first two or three days it is replaced by a yellowish tint, we must make a minute examination. To this circumstance we shall recur presently.

Paleness being one of the most remarkable phenomena in other serious diseases besides œdema of the cellular tissue, we shall speak of it when we come to the diseases in which it occurs.

*Expression of the Face.*—The countenance of a new-born child, when it is not suffering, is devoid of expression. This however is true only of the new-born child, and is not applicable to a child at the breast. Even when a month old, the child, from being in good health and from its functions being duly performed, expresses by the very insensibility, if I may so say, of its features, this state of well-being, which disappears only to be replaced by an expression of suffering, when it feels a desire for the breast, or when any of its functions are not duly performed. This insensibility, or inappreciability alluded to, is the intermediate state between retraction of the features, which is an expression of pain, and their dilatation which constitutes smiling, and is an expression of pleasure. Immobility of the features constitutes an intermediate state between this inappreciability above alluded to and painful contraction. This has been found to be occasioned chiefly by milk of bad quality, or insufficient in quantity.

When the countenance of the new-born infant is immovable and without expression, the mouth is closed, and there is no wrinkle between the brows. But as soon as there is pain, changes supervene which, by their continuity or intermission, indicate intermission or continuity of the pain itself. In acute affections there is contraction of the features, numerous wrinkles on the forehead; approximation of the brows, which are cut perpendicularly by numerous furrows, the commissures of the lips are drawn outwards; in a word, the face is now sharpened. At a more advanced period of disease, the contraction of the features becomes permanent, and gives the child the appearance of old age, owing to the form of the dental arches destitute of teeth at the two extremities of life. According to Billard, the *pain-expressing countenance* of the new-born infants, like that of adults a prey to violent pain, consists in the upper lip being wrinkled and half-raised; vertical and horizontal wrinkles are observable at the root of the nose, and extend to the forehead; the eye-lids approximate, and several wrinkles form at the external angle of the eye, or are marked circularly in the skin. We conclude our remarks on this sign by observing that every modification of the countenance which shall exhibit a trace of pain more or less marked will always be a sign of disease more or less advanced.

We shall continue this subject in our next.

---

ELEMENTS OF CHEMICAL ANALYSIS, INORGANIC AND ORGANIC. By *Edward Andrew Parnell,* Chemical Assistant in University College. London, Taylor and Walton, 1842.

THE rapid advances which the science of practical chemistry has made within the last few years are truly extraordinary. The untiring industry and indefatigable zeal of Liebig, Rose, Berzelius, Orfila, and others on the Continent, as also of Thomson, Turner, Graham and Kane in our own country, have given an entirely new aspect to this important and valuable branch of natural philosophy. The interesting discoveries of Liebig more especially in animal chemistry, of which we have given a resumé in the last number of our Journal, cannot fail to attract the attention of physiologists and pathologists. The work now before us, the object of which is to give rules and directions for conducting the manipulations of chemical analysis, coming from a gentleman, who enjoys such peculiar advantages for attaining a perfect knowledge of his subject, will be found a valuable contribution to chemical science. It consists of four Chapters. The first contains plain instructions for performing the ordinary manipulations of analytical chemistry, with a description of the most important tests used in chemistry, and the means of detecting the impurities with which these tests are occasionally adulterated. The second chapter contains an account of the appearances produced by the principal re-agents, when applied to various chemical substances. In this chapter will be found the use of the mouth blow-pipe in the manipulation of chemical analysis. The third chapter contains some excellent directions for conducting the qualitative analysis of compound substances. In this chapter will be found some excellent directions on the use of the blow-pipe in qualitative analysis, on the analysis of gases, and on the detection of poisons in organic mixtures. A subject of serious importance to medical men, viz. the qualitative analysis of urine, and the discrimination of urinary calculi, is contained in this chapter. Quantitative analysis forms the subject of the fourth and last chapter. Organic analyses, and the determination of the nitrogen contained in organic bodies, will be found explained with great clearness. An Appendix has been subjoined containing the method of calculating the atomic constitution of a body from its per centage composition, as also the various methods of taking the specific gravity of bodies. We have perused this interesting volume with great satisfaction, and have no hesitation in recommending it strongly, as a safe and valuable guide in conducting the intricate and perplexing operations of analytical chemistry.

---

Thesaurus Medicaminum: or the Medical Prescriber's Vade Mecum; containing all the Medicinal Substances of the Pharmacopœia, arranged according to their Therapeutic Action; with the most elegant Method of Prescribing each; and a Table of Incompatibles, and Directions for the Treatment of Poisoning. By *D. Spillan,* M.D. Baillière, Regent-street.

The name given to this little book reminds us of a book of the same name, and of a similar description, written by Dr. R. Pearson many years ago. The object which the author had in view in compiling this little work was to enable the junior practitioner to obtain at a glance a knowledge of the therapeutical action of the various substances employed in medicine, of those preparations of them which are officinal in the British Pharmacopœias, and of the method of combining them in extemporaneous prescription. What strikes us as novel in this work is, that to almost every article, whether simple or compound, a formula is subjoined, illustrative of the most elegant method of ordering it in prescription. The prescriptions are decidedly excellent and effective, and contain all the newest remedies. The great advantage of a work, like the present, consists in this, that it presents to the medical practitioner a bird's-eye view of all the implements of his art, whereby he is saved an immensity of trouble and of time. The Table of Incompatibles is drawn up in such a manner that it cannot fail to prove of considerable use in preventing those errors of chemical combination so often destructive of medicinal efficacy in the best articles of the materia medica. We have no doubt but that Dr. Spillan's little "Thesaurus" will prove extremely useful to the practitioner. We feel satisfied that to urge the book on the notice of the profession it requires only to be seen in order to be appreciated.

---

Report of Her Majesty's Commissioners on the State of the British Settlements on the Western Coast of Africa. By *R. R. Madden,* M.D.

### Climate and its Influence on Health.

The instructions given to Dr. Madden as Commissioner of Inquiry on the Western Coast of Africa, by the Secretary of State for the Colonies, Lord John Russell, required of him to investigate the climate, general capabilities, commercial resources, and military advantages of the different settlements, as also to report on the state of the slave trade—the prospects of emigration from Sierra Leone to the British West India Colonies—and generally an enquiry into the moral and physical condition of the native population.

How ably and usefully Dr. Madden has executed these orders, the pages of his excellent "Report" fully attest; and were it not that the subject of climate —the interesting and all-important subject of climate—is too intricate and delicate for the generality of our practical English readers, we should take pleasure in quoting largely from Dr. Madden. We hope to do so on a future occasion, meanwhile, we can assure our brethren that, the best practitioners anywhere to be found will be those who have studied climate and disease in all their varieties. In temperate climates, all other circumstances being equal, the best physicians we are acquainted with are those who have known the worst climates with the most fatal and concentrated forms of disease belonging to them. To these it seems easy to contemplate the lesser degrees of diseased action, and to treat them with effect. It is thus that an acquaintance with unhealthy regions, becomes of practical utility to the British physician. The subject of the external causes of disease forms a department of itself, second in importance to no other that we could name; and we refer with pride to the almost exclusive investigation and elucidation of these causes by the medical officers of our fleets and armies, many of whom have subsequently carried their knowledge and experience, greatly to the benefit of the community, into our cities and counties.

The report of Dr. Madden comprehends :—"The general Climatorial Character of our different Settlements—Observations on Professor Daniell's Report on the Waters of the African Rivers—Climate of Sierra Leone, of Gambia, of the Gold Coast—Bilious Remittent, or Seasoning Fever—the Question of Contagion —the Influence of Climate on the Manners, Laws, and Religious Observances of the Natives—Mortality from Fever at Sierra Leone, &c.—Mortality at Bulama —Maxims on the mode of Living in Tropical Climates—Rules for the Preservation of Health in Hot Climates—Meteorological Journal." On all these points the reader will find important and interesting information; but we regret we cannot now enter upon them. Dr. Madden confirms the accuracy of Major Tulloch's Statistical Reports, although, he says, "the facts therein stated are called in question in our African settlements ;"—indeed, we find that here, as in others of our worst climates, the surviving Europeans are under a strange and gross delusion as to their influences—extenuating the disadvantages of their respective stations—so as to deceive others, "by dint of endeavouring to impose upon themselves, and to establish the opinion that, whenever a man dies, the climate is not in fault, but his own constitutional peculiarities, or imprudence of some kind." Whoever has sojourned in tropical climates will remember this continual self-delusion on the part of the white inhabitants.

Of eight witnesses to the best modes of treatment in the fevers of the West Coast of Africa, seven recommend the use of mercury to salivation, and the eighth uses it in a more restricted manner—" only as a purgative." Bloodletting is practised with caution, and only by a few of the practitioners even locally.

Five out of the eight do not even mention the abstraction of blood. Quinine is very generally administered during remissions.

"The most successful practice, however," says Dr. Madden, "that any where on the Coast of Africa came to my knowledge, was that I received an account of, from the Dutch authorities at Elmina, of the system of treatment pursued by the surgeon of that settlement, and here mercury in any shape was never had recourse to, nor the employment of the lancet, but leeches largely over the stomach, and to the temples, forty or fifty at a time, and repeated so long as there was pain or pressure over the epigastrium. Quinine subsequently on the disappearance of the fever is exhibited externally, strewed over the raw surface left after the application of a blister on the stomach."

Without statistical and detailed statement of comparative result, it were vain to offer any opinion on a practical question of this sort. We cannot, however, help observing, in reference to the claims here set forth, and to the careful exclusion of "the lancet," that a fever and a subject that could bear "40 or 50 leeches at a time, and repeated as long as there was pain on pressure over the epigastrium," must be, the one, anything but of a low type, and the other, anything but feeble; for, in the generality of the low congestive tropical fevers, the application of leeches in such large numbers, even without subsequent oozing, has often appeared to us to sink the patient more than a moderate and well-timed abstraction by the lancet.

But it is in the time of using it that great errors and mistakes have been committed in regard to blood-letting in tropical fevers, and that so much misapprehension has arisen as to its utility. It is a point, however, that requires the greatest power of observation, experience, tact, and judgment.

---

I. A System of Practical Surgery. By *William Fergusson,* F.R.S.E. Professor of Surgery in King's College, London. Post 8vo. pp. 595, Churchill, London.

II. Principles of Surgery. By *James Syme,* F.R.S.E. Professor of Clinical Surgery in the University of Edinburgh. Third Edition, enlarged, 8vo. pp. 508. H. Baillière, London.

Although surgery makes less rapid strides than many sister arts and sciences, so that we do not open a new work, as we might a treatise on chemistry or physiology, half prepared for a completely new series of facts and a revolution in theories, since the last publication on the same subject, yet its progress within the last twenty years has been sufficiently remarkable to be worthy of observation.

The Scottish School of Surgery and its leading characteristics, in contradistinction to those of the English and Continental Schools, may have glanced through our mind on reading the titles, and the names of the authors, both distinguished Professors of Edinburgh, and one of whom but lately carried away from English competitors the Chair of Surgery at the King's College. But on closing the work of the latter we found we had wandered over the whole field of surgery—marking its progress, not in one School alone, but in Europe, during the last quarter of a century, and the gradual change of precept and practice among the practitioners of the day. If somewhat surprised that an elementary work, for the instruction of students chiefly, in practical surgery, should have led us so far, we also felt that Mr. Fergusson had obtained the best compliment we could pay to the merits of his book. Had he not treated of surgery as a science, and avoided the merely conventional doctrines of a particular school, we should scarcely have accompanied him through many pages.

He leaves no principle of practice of real interest untouched, but with an easy and perspicuous style brings before the reader all essential facts, and the doctrines founded upon them, and while stating his own opinion, never fails to weigh with fairness and impartiality the views which others, entitled to consideration, entertain on the same subject.

Such a work we conceive has long been a great desideratum, not only to the student, but to the practitioner. Mr. Cooper's Dictionary may give all that is required, but it gives so much more, that if it do not altogether repel the student, it leaves him bewildered and confused. The same gentleman's "Elements," however excellent, is wanting in much as a practical work. The same observation applies to Mr. Liston's "Elements," while his admirable work on "Operative Surgery" is dedicated exclusively to that division of the art. Burn's Elements, are elementary in the heaviest sense. Mr. Syme's Principles of Surgery, (the third edition of which is now before us) presents many advantages over these, and although enlarged is still not too bulky—but it has the drawback of being a description of the practice of one surgeon, and the doctrines of one school, rather than a general review of the principles of surgery. It treats many of the most important points with a Spartan brevity. Lastly, we must not omit the very clever, but withal, vade-mecum style of work by Mr. Druitt, where too much and too little are alike attempted, for a really practical work on surgery. We do not consider Mr. Fergusson's book perfect, or without objections; we are inclined however to think that it goes far to supply the deficiency experienced by the student in following the lectures of his teacher in surgery, and will moreover form a very valuable little work to the practitioner for ready reference. The woodcuts are in Mr. Bagg's best style, a great acquisition, and worthy of all praise for their spirit, clearness, and accuracy.

Mr. Fergusson states his own design in the production of this volume to have been "to produce a manual of the details of practical surgery, which shall, in some degree, meet the wishes and wants of the student, as well as of the surgeon already engaged in practice, and it is hoped that the volume may prove of some value to both parties."

"The present work has not been produced to compete with any already before the profession,—the arrangement, the manner in which the subjects have been treated, and the illustrations, are all different from any of the kind in the English language."

The work is divided into five parts, and the first of these, consisting of some 120 closely printed pages, is devoted to the "Elements of Practical Surgery." To this section we would particularly direct attention, as full of sound principles, and excellent precepts for their practical application, with much that is either not stated so well, or altogether omitted, in other elementary and practical works.

Here is our author's definition of practical surgery:

"Under the term Practical Surgery, I shall include the symptoms of disease and injury, the principles and objects of treatment, and such medicinal means of cure as seem to me to belong to the province of surgery; and under what is commonly called Operative Surgery, I shall describe not only those operations which are performed with cutting instruments, but also the various duties required on the part of the surgeon, as in the setting of fractured bones, reduction of dislocations, application of bandages, and other manipulations of Practical Surgery."

There is much good sense in the following appreciation of ambi-dexterity.

"It will be of advantage to dissect occasionally with the left hand; but there are few who will attain the same command over it as over the opposite one. From infancy we apply each hand to special purposes; the left hand has its peculiar duties to perform whilst the other is engaged,—it may be said to be the servant of the right; and on the part of the surgeon this seems to me all that is necessary; for it appears as absurd to expect ambi-dexterity with him, as it would be to expect, or recommend it, with the painter, sculptor, or common mechanic. I have never yet seen a surgeon who possessed equal power and grace in either hand, nor do I consider that the efficient practice of his art requires that kind of dexterity possessed by the juggler, who tosses his daggers, and catches them again as they twirl through the air, as freely with the one hand as with the other, and with whom the movements of each hand are, on such occasions, of necessity, nearly alike."

Students would do well to act upon the advice the author gives, and his estimate of the importance of the minor duties of a surgeon.

"There cannot be a greater mistake in a young surgeon's education, than to commence the performance of operations before he has acquired a thorough knowledge of anatomy. If he enters into practice without such knowledge, he can neither operate with safety to his patient nor satisfaction to himself; all must be hap-hazard; whilst on the other hand, in prosecuting his dissections, he takes the surest way of acquiring that dexterity in the use of his hands and instruments, which will be of infinite service to him afterwards in the ordinary performance of his professional avocations; and here I do not so much allude to the performance of capital operations, as to the more common manipulations required in bloodletting, bandaging, dressing sores, opening abscesses, and the numerous little manual proceedings which constitute the routine of surgical practice, and which, though seldom named as 'operations,' should be deemed as characteristic of good practical surgery, as the dexterous removal of a limb, or the rapid and successful extraction of a stone from the bladder."

Passing in review the different instruments in use, our attention is called to changes which have taken place as to their selection and uses :—to some, which although among the oldest known, are new in the general, frequent, and beneficial application made of them in late years. As often may be observed, the most valuable are not those where the ingenuity has been put upon the rack to combine in one instrument all the mechanical powers, but some of the simplest form and character.

"The cutting forceps may be used for the removal of portions of bones, when the saw cannot be readily applied; and in some instances, as in excision of portions of the jaws, this instrument may be substituted with great advantage; there is but little skill required in using it, strength of hand being the chief requisite."

"For the removal of diseased or dead portions of bone, when the neighbouring healthy bone requires to be cut, it may entirely supersede the common saw, or the more complicated one, termed the chain-saw. Indeed, though this is a somewhat ancient surgical instrument (for it was figured by Scultetus nearly two hundred years ago), and though it had become in a manner obsolete till within these thirty years, I know of no single instrument whose re-introduction to practice in modern times has conferred a greater boon on the operating surgeon; and for this I believe we are solely indebted to Mr. Liston, whose example in using it has been most extensively followed by a large portion of the present race of operating surgeons."

Mr. Fergusson gives much praise also to the gouge, which he observes has been in a great measure overlooked by the modern surgeon. In operations for necrosis and caries he deems it quite indispensable. In such cases (rare we must believe them to be) where it may be expedient entirely to remove diseased surfaces of bone by violence, we are not inclined to quarrel with the gouge as a simple and efficient instrument for the purpose.

The author's directions as to the preparatory arrangements for operations are excellent, and his recommendation, that the operator should examine, and assure himself, that every thing he may require is at hand, and in a good state, is well worthy of attention, if surgeons would avoid the dilemma of an operator referred to by Sir Anthony Carlisle, who, after dividing the soft parts of a limb, had to send for a saw many miles off!

A little attention to these details, too often considered beneath the notice of our hospital surgeons—and to the preliminary arrangement among the assistants of the specific duty each should undertake, would not only tend much to the decency and celerity of operations and the welfare of the patients, but above all, would prevent scenes of noise and confusion, with their attendant loss of time, but too frequently seen. The London hospitals in these matters reflect but little credit in general upon English surgeons, and are far behind either the Edinburgh or Continental hospitals, and although Roux could not now with equal readiness find an hospital surgeon consuming twenty minutes in removing a limb—yet similar instances of tedious and bungling operations may too frequently be seen in more than one of our Metropolitan hospitals.

Under the head of "Counter-irritants," there are some very good observations, written in a fair and candid spirit; he thus sums up.

"Of these three modes of counter-irritation, I give a decided preference to the last. The caustic potash (the potential cautery, as it is usually termed) produces its effects far more slowly, and with less certainty than either of the other two, being in some instances apt to go deeper than might be wished, or to spread too extensively over the surface; and it is chiefly on the latter account that the plaster with the hole in it is recommended, as a preventive. The moxa is troublesome to apply effectively; and often, after the patient has been put to a deal of pain, its results on the surface are so limited that the counter-irritation is slight indeed. My old colleague, Mr. Lizars, used the potash most extensively, and the groans and stifled screams of agony heard in his wards a few hours after his visit, bore ample testimony to the pain which it produced: I have myself used it frequently, and, of course, must plead guilty of the same kind of cruelty. The moxa, I believe, produces much more acute pain than the caustic, and possesses no advantage over it that I am aware of. At one time I used this remedy pretty often, but for the reasons above stated I have latterly almost entirely given it up; and now, when I have the power of selection, I almost invariably resort to the heated iron."

"Though there are still many prejudices against it, and certainly many just objections to its indiscriminate use, it seems to be more in vogue among modern surgeons than it once was; and, under judicious guidance, it appears to me a more manageable, equally efficient, and less painful counter-irritant than either of those last mentioned. It is, besides, highly serviceable in arresting hemorrhage, when no other means will answer, and is altogether of much use in surgery, as will be explained more particularly afterwards. It must be confessed, however, that there is an appearance of rudeness and cruelty in its application, which must always render its frequent use objectionable in modern practice."

The application of the cautery over a large surface with intervals, so as to avoid a slough—and the beneficial action of this method upon chronic cases of diseased bone or periosteum, are at once judicious and practical.

"Instead of applying a ball or single mass of iron, so as to cause a slough on the surface, an inch or more in length, and perhaps half the breadth, I generally bring a larger extent of surface under its influence, by scoring the skin much in the same way as the operation of firing is done in the horse. The process is slower, and therefore more painful than a single touch of the instrument, but in some cases it presents such advantages, in my opinion, that, when the cautery is actually appealed to, the difference may be overlooked. I was first led to apply it in this way, from drawing some analogy between certain nodes on the tibia and splints on the young horse. In such instances it is well known that, when the animal continues lame for a length of time, and when stimulating ointments and blisters have seemingly had no effect, the application of the cautery effects a cure, more particularly if due rest is given afterwards. Although in most instances a painful node requires constitutional treatment as well as local, and differs in almost all respects from what is termed a splint in the lower animal, yet occasionally painful chronic swellings are met with on the surface of the tibia, which have resisted all the ordinary modes of treatment, internal as well as external. Many years ago a case of this kind occurred in my practice. A young man, aged twenty-three, had two years before contracted syphilis, and after the primary symptoms were cured, a node formed on the tibia, which occasioned him much pain:—from being a person of stout appearance, he now seemed like one exhausted by a painful and malignant disease. Before I saw him he had undergone various courses of mercury and other medicines, and had the part repeatedly leeched and blistered. I again subjected him to similar practice, and also tried iodine freely, both internally and externally, but with no mitigation of his sufferings. Rest seemed to produce as little benefit, and his disease was worse at night. For six months I watched the progress of the case, and then I applied the cautery in the mode referred to. During the evening he slept better than he had done for three months before, and from that time his disease was cured. I saw him occasionally for years after, and he had never subsequently experienced another twinge of pain in the swelling. I have had similar success in other examples; and though I would not wish any one to imagine, that I predict or would anticipate invariable success from the measure, I cannot but strongly recommend it, in such-like instances, especially where the affection seems to be, and perhaps is, entirely local."

"The chief advantage which I fancy the plan above described to possess is, that sudden excitement is produced over a large surface, whilst at the same time the skin is not destroyed and converted into a slough, as with the caustic or moxa, so as to leave a large open surface, which will heal but slowly, and will always leave a considerable scar. If, on the other hand, a round or flat piece of iron is applied even once to the surface, a slough must be produced, which separates more slowly, and leaves a more troublesome sore than the small lines of sloughs, and the consecutive sores, resulting from the practice above recommended."

Among the modern improvements of surgery, apparently trifling, but in reality important, we believe cold and warm water dressing may deservedly be placed very high in the list—were it merely for the exile of poultices, or "cover sluts," as they have been not inaptly termed, which it is their tendency to effect, the practical benefit would be great.

We cordially concur in the praise given by our author to this means of reducing inflammation.

"If I deemed warmth required, instead of using a poultice, I would wrap the part up in wet lint, and cover it with oiled silk. Moisture and heat are thus more effectually secured than by a poultice, which will soon get cold on the surface, and in a few hours become almost quite dry. On other parts of the body this plan, modified by circumstances, may be resorted to with every advantage, and I cannot speak in terms too laudatory of the 'warm-water dressing,' as this method is now usually called, which, under the influence of many practical surgeons of the day, has in a great measure superseded the more troublesome, and more cumbersome poultice."

In treating of the consequences of inflammation and the appropriate treatment, we find much to recommend, although little that is absolutely new. The observations on shivering as a diagnostic sign of suppuration—pointing out its uncertainty, are good, as also on the proper period for giving exit to matter.

Seeing the judgment and impartiality with which Mr. Fergusson handles all the leading questions in surgery, we have been induced to give more attention to these subjects than a work of this kind usually demands or repays—we will endeavour very cursorily to present our readers with the result.

QUESTION OF AMPUTATION IN GANGRENE AND MORTIFICATION.

We must confess our own experience of the propriety of amputating before an obvious and vigorous effort at separation has completely isolated, to some depth, the dead portion, although it furnishes several successful cases, is, upon the whole, unfavourable to early amputation and its risks—our author takes a similar view. He says,

"In the instance of spreading gangrene, as has already been stated, it is difficult, if not impossible, to say where the disease is to end,—where there is to be a separation between the dead and living parts,—and hence it has been the prevalent custom to wait until a line of demarcation has formed, though, from the examples of Larrey, Lawrence, and some other modern surgeons, the practice of operating at an early period has been strongly advocated. Although educated in these latter doctrines, and strongly prepossessed in their favour, I feel bound to say, that, after having acted upon them repeatedly, and seen others do the same, the success has been very different from what I anticipated. I have in my recollection six cases in which I amputated during spreading gangrene, four times in the thigh, (one of them being for a simple fracture of the leg, another for compound; both close upon the ankle; the third following spontaneous obstruction of a popliteal aneurism, and the fourth after ligature of the femoral artery for a similar disease); one in the leg for severe lacerated wound of the foot, and the sixth at the shoulder-joint for extensive injury of the arm. None of these succeeded; and though I might possibly in future resort to similar practice, I must say, that I should feel greatly inclined to wait for a line of demarcation; and, that even here, I should not be very sanguine as to the result. Numerous cases might be brought forward, however, to prove the success of such practice, yet I believe that, in many instances, the surgeon will best show his judgment, by amputating, in severe injuries, before sufficient time has elapsed for gangrene to come on, or by waiting, in the event of such an occurrence, until it is seen how far, and to what degree, the affection is likely to proceed, and in addition, to what extent the constitution sympathises with the local disease. The latter circumstance is, indeed, often remarkable; but whether it is from the wound or the gangrene, it is difficult to say. I once saw an amputation in the leg performed by a surgeon of great experience, for a severe compound fracture: the calf of the leg, when the incisions were made, was in a slightly suspicious condition, but not sufficient to deter from selecting this part for the operation: unequivocal gangrene, however, attacked the stump, and within eight-and-forty hours amputation was performed in the thigh: again the disease appeared in the stump, and at the same time in the skin over one of the scapulæ, where there was no suspicion even that the slightest injury had been inflicted."

ERYSIPELAS.

The author, while very justly, in our opinion, reprobating the abuse of incisions—extending, as we have too often seen them, in one continuous line, from the trochanter major to the malleolus—so that on raising the limb, the whole external covering of skin and cellular tissue hung pendent like a wrapper of chamois leather—we think he underrates their free use within more judicious limits. In many cases, we are satisfied the worst results have been averted by three or four incisions down to the seat of the sloughing and suppurative action, of from three to six inches in length, if the whole lower extremity be involved.

FALSE JOINTS.

Mr. Fergusson is less adventurous—and we may add more judicious, than our brethren across the Atlantic in reference to the operative means for remedying this mischievous consequence of fracture. There is fortunately no great divergence of opinion among our most distinguished surgeons upon this class of cases.—He says,

"My opinion regarding false joints, whether resulting from fractures or dislocations, will be found at greater length in future pages, where I shall speak of particular cases; meantime, I may here state, that in the treatment of non-union, I would resort to almost any reasonable measure before I would cut down to the parts, as was done by White."

"I would only cut down to the fragments as a last resource, and, on doing so, be regulated by circumstances, whether I should merely scrape the exposed surfaces, or actually cut portions of them away with the saw. In the leg or fore-arm, I should resort to the latter plan with great reluctance, more especially if only one bone was at fault."

We saw, not very long ago, a case of failure in one of the London hospitals—where the humerus was cut down upon, and after a most trying and tedious dissection to detach the slightly oblique edges of the fracture, extensively united by ligamentous and cartilaginous structure, from each other—with some violence, one end of bone was brought out and removed by the saw.

None but a robust man would have been likely to have borne this operation without imminent danger from its constitutional effects—and the result satisfied us—that an infinitely nicer adaptation of means, and more assiduous care and gentleness to secure coaptation and immobility would be in all cases required, to offer a reasonable chance of success in a public hospital.

We cordially agree with Mr. F. that "any reasonable measure"—were better. He gives an instructive example of the benefits of full doses of whiskey, in a habit previously debilitated, where it had formed the customary stimulus, and the influence the treatment exercised on the process of union.

EXCISION OF JOINTS.

This subject Mr. Syme has made his own nearly, and we observe with pleasure the truly rational and judicious view he takes of the applicability of this operation.

"Amputation has until lately been regarded as almost the only means of relief from carious joints. But it is now ascertained by experience, that, on some occasions, the limb may be saved by cutting out the articulation. The softened, discoloured, and ulcerated integuments, the thickened and indurated cellular substance, and the gelatinous synovial membrane, are found to afford no serious obstacle to recovery, provided the whole of the bone, so far as it is actually carious, be taken away. The operation requisite for this purpose, though severe, is not more dangerous than amputation, because the joint, previous to its performance, has been opened by the disease; the whole of the articulating tissues which are apt to suffer violent inflammation when irritated are either previously destroyed or removed; the great blood-vessels and nerves are not interfered with; and the patient is not subjected to the shock which is caused by taking away a limb.

"As to the joints which may be subjected to this operation, it is evident that the extent to which the acetabulum is almost always affected in the hip-disease forbids any attempt at excision. Though experience has not yet fully decided whether the limbs that might be preserved by cutting out the knee and ankle-joints would be preferable to the artificial substitutes which may be worn in their stead, it seems pretty well ascertained that they would not. The wrist, also, from the number of bones and complexity of articulations entering into its formation, together with the numerous tendons, arteries, and nerves passing over it, does not seem to be within reach of the operation. But the elbow and shoulder-joints, while their structure and situation are most favourable for excision, hold out the greatest inducements to effect their removal without performing amputation. In all ranks and circumstances of life, the use of the hand is of great consequence, and though the elbow or shoulder were to remain perfectly stiff and motionless, yet, if the hand could be preserved entire and serviceable, by excision of these joints, it would be infinitely preferable to do so, instead of taking away the limb. But it has been proved by numerous facts, that while the joints beyond the disease remain as useful as ever, the one which has undergone the operation regains such a degree of mobility and subjection to the action of its muscles as sometimes to render it hardly distinguishable from a sound one, and in general prevents it from at all impeding the use of the arm by its stiffness. There is no new joint, strictly speaking, formed, but a strong fibrous substance unites the extremities of the bones, and by its flexibility allows them to move within proper bounds; while the muscles cut across in the operation obtain new attachments, so as perform their usual office."

As Mr. Syme's experience in this operation is probably greater than that of any other surgeon of the present day, his opinion is nearly decisive.

### AMPUTATIONS.

Primary or secondary—flap or circular—short cut ligatures or long—torsion or ligature—immediate or deferred dressing—these are all questions of interest and importance, which (with the exception of the first) had been little discussed until the last few years. On some of these points, surgeons are nearly unanimous, while, on others, considerable difference still exists.

*Primary or Secondary?*—" In all instances," says Mr. Fergusson, " when a limb is injured beyond the hope of recovery, there seems little doubt among the present race of surgeons, that immediate or primary amputation should be performed; the statistics collected by Mr. Guthrie, and still more recently by Mr. Rutherford Alcock, give decided proof in favour of this practice."

This does not altogether coincide with the impression Mr. Alcock's publications and lectures on this subject have given us, and on referring to the concluding lecture of the series which appeared in the Lancet, giving a summary of his views, we find that he makes a distinction, founded upon statistical results, between the injuries of civil and military life—and by no means regards primary amputations in civil hospitals as the most advantageous. We find it stated on the contrary that—

" The results of military and civil hospitals combined stand in the following order:—

" First. Amputations for chronic local disease.

" Second. Primary amputations for injuries of military life.

" Third. Secondary amputations for injuries of civil life.

" Fourth. Primary amputations for injuries of civil life.

" Fifth. Secondary amputations for injuries of military life.

" The question of primary and secondary amputation is here reversed in military and civil life. The main doctrine on which the army surgeons explained and upheld the superior excellence of primary amputation and its universal application (viz. the rude health of the patient), is shaken to the centre by the most successful of all the series of cases proving to be those performed where long pre-existing disease, and often confinement, has removed the patient far from a state of robust and plethoric health, to one of debility and emaciation.

" Is it not strange that the medical practitioners of civil life, of large cities, whose talents are employed in large institutions, where hundreds of cases of amputation for injury and disease, in a few years must pass under their observation, should for so long a period have accepted doctrines and results, the first fallacious, and the latter inapplicable, and to which the facts and experience of their own practice are totally opposed?"

As the latest writer, who with much experience, has devoted great attention to this subject, his conclusions demand consideration—if found correct, they would inevitably lead to a great change in the opinions more generally held on the subject of primary and secondary amputation.

*Mode of Operation.*—Nothing can be more impartial than the estimate we find in Mr. Fergusson's work of the relative value—the advantages and disadvantages of the flap and circular operations. We recommend it to all practitioners who may not have the means of forming their opinions from extensive opportunities of watching the performance and results of the two plans.

Mr. Fergusson's opinion is, that in skilful hands there is no very great or preponderating advantage on either side, and this, from one brought up in the school of flap operations, is strong evidence. We regret we have not left ourselves space for any extracts to do more justice to the able manner in which the author has handled this part of his subject.

Of torsion of arteries as a substitute for ligatures, it is truly observed that it never established itself among surgeons generally, and with only a very doubtful advantage at best, it presents many very serious drawbacks.

In reference to deferred dressing of the stump, we are glad to find that Mr. Fergusson does not "hesitate to recommend that, as a common rule, the wound of an amputation should be dressed while the patient is on the operating table." Indeed the deferred mode of dressing, like the torsion of arteries, has never been established among English surgeons, and they may be classed together among the modern attempts at improvement that have been finally condemned. We must not however go farther into this subject—and can indeed only glance at the remaining parts of these works.

In the sections devoted in both to the injuries of the head we cannot avoid feeling that this subject has been but superficially handled, and that they imperfectly represent the present state of surgery and opinion, in reference to these most interesting and important injuries. The same observation applies in a lesser degree to the injuries, wounds, and operations of the chest and abdomen.

A large portion of Mr. Fergusson's work is devoted to a consideration of the operations required by injuries and disease—on which little need be said. The instructions given are clear, precise, and well adapted to attain the object of the author, while nothing can be more perfect than the plates in illustration, which are sketched with a masterly hand, and cannot fail to be of great assistance in rendering the description more vivid and easy of comprehension.

We had intended giving to that section of Mr. Fergusson's work devoted to the subject of lithotrity and the relative merits of lithotomy more space than we have left ourselves. The following extracts we may safely recommend to the consideration of our readers, as a resumé of the whole.

"Like many other novelties, lithotrity has undoubtedly been too much vaunted by its professed advocates and performers; but it is equally clear that in many instances it forms an admirable substitute for lithotomy. Notwithstanding the reputed success of Civiale, it seems to me that, in the present stage of its history, we have not sufficiently authentic data by which to determine the comparative safety of lithotrity to that of lithotomy; but regarding the applicability of the former, and even its superiority in many instances, there need be no doubt. Years must yet elapse, and the operation must be tested in our public hospitals by the same class of surgeons as those on whose proceedings the statistics of lithotomy have been founded, before an unbiassed professional judgment can be given on the subject."

"But from my own experience I should say, that the most formidable objection to lithotrity is the apparent irritability of the urinary organs: if the patient does more than wince while being sounded; if the application of the steel to the urethra seems to occasion pain—I mean more than that sensation which patients usually have on such occasions—if the mucous surface of the bladder is so tender as to cause the contact of the instrument to be borne with difficulty; and if the muscular fibres are excited to such violent contraction as to occasion the evacuation of the fluid contents along the side of the instrument, or to excite an irresistible desire to micturate, then assuredly the circumstances are peculiarly unfavourable to the proceeding."

"Although lithotrity may be performed on children, it may well be doubted if such a proceeding should ever be attempted upon them; for it would be difficult to name any single operation of magnitude which has been more successful on young subjects than that of lithotomy. Out of one hundred and five cases operated on by the latter method in the Norwich Hospital—the patients being under ten years of age—only three died, thus giving an average of one in thirty-five: and although other tables do not show altogether such favourable results, there are good reasons for supposing, that the average deaths in young persons who are subjected to lithotomy is little more than one in twenty-eight or thirty. Until it can be shown, then, that lithotrity surpasses this success, and is in almost every other respect to be preferred, it is only a fair conclusion to draw at the present time, that lithotomy is decidedly preferable in such subjects; and when, moreover, the comparative frequency of the disease in children is taken into account, it will at once appear that a large proportion of all cases of stone must yet be set aside for the lithotomist. Above the age of puberty, however, the average alters very materially, and, as already stated, the propriety of resorting to lithotrity ought to have due consideration."

In reference to this last observation we had forcible proof of the danger of exciting any great irritation by the passage of instruments, in a little boy who came under our observation a short time back. He had a small stone in the bladder, detected once by the surgeon in attendance, but not subsequently, and two surgeons of eminence sounded in vain upon two or three occasions. In the course of three weeks all doubt was solved by the death of the little sufferer from the irritative fever induced. It ought to be received as an axiom in surgery—*Below the age of puberty lithotrity is not applicable.* In maturer age the question is yet to be decided, and not by every clumsy bungler who can push an instrument into the bladder; but by those whose skill, tact, and delicacy of touch will leave no doubt as to the fairness of any results following their operations.

The new edition of Mr. Syme's work, is not only enlarged but improved; and, in reference to Mr. Fergusson's book, we sincerely congratulate the students in the possession of so able a guide to the study of surgery, and feel assured it will speedily recommend itself very generally, not only to students but to practitioners, for whose use it appears not less specially designed.

---

REPORT ON THE CHIEF RESULTS OBTAINED BY THE USE OF THE MICROSCOPE, IN THE STUDY OF HUMAN ANATOMY AND PHYSIOLOGY. By *James Paget*, Demonstrator of Morbid Anatomy at St. Bartholomew's Hospital, &c. London, Churchill, 1842.

It does not consist with the spirit or the habit of this Journal to go deeply into researches that are obviously barren of practical application. Microscopic inquiries have hitherto fallen within this category, and we fear they are still likely to be retained in it. We cannot therefore examine at any length the Report before us, nor present our readers with anything like an elaborate account of it. But it would be the height of injustice to pass it over in silence, or to neglect the opportunity of awarding to its author, Mr. Paget, the praise he richly merits, of research, accuracy, and ability. It is a very admirable summary of the present state of our knowledge of the miscroscopic structure of the body.

The ground-work of all—the *Nature and Development of Cells* may be laid before our readers. We are sure they will read with interest, if not with actual profit, Mr. Paget's epitome of Schwann's views. Condensed, itself, to the utmost, further condensation is next to impossible.

To Schleiden and Schwann is due the discovery of the laws of development of and through cells. These laws are invalidated somewhat in their details by late investigations, but confirmed apparently in their general bearing. We shall, without further preface, state them.

1. *The Cells.*—" A Primary Cell, or as it is sometimes called, a nucleated or elementary cell, is a minute vesicle, usually of a spheroidal or oval form, composed of a fine transparent membrane, containing an albuminous, gelatinous, oily, granular, or other fluid, and having on its wall, or in its interior, a small body called the nucleus or cytoblast, which, again, commonly contains one or more dark round spots, nucleoli, or corpuscles. Such cells are found either persistently or temporarily, as one of the forms passed through in their development, in all, or nearly all, organized tissues, whether animal or vegetable, normal or morbid. Whatever be the tissue to be developed, the first periods of its development seem to be similar. There is not first developed for each structure a peculiar form; nor is each produced first on a small scale and then enlarged: but in all, the molecules are first arranged according to similar laws; and the nucleated cell is the form up to which in every tissue the principle of development seems to tend, and from which it proceeds in a different direction for the further development of each."

2. *The Cytoblastema.*—" The material in which the cells are formed, and from which they derive their means of increase, is a structureless fluid or soft substance, and is named cytoblastema. In the embryo it is that which has been called the formative, or primary, or indifferent substance: in the perfect animal it is the nutritive material effused from the blood-vessels into the interstices of already-existing tissues. When cells are formed and fixed in it, it is usually called intercellular substance, and may undergo changes which contribute much to the character of the fully-developed tissue."

3. *Development of the Cell.*—" The development of the cell is attained in different methods, but the chief of them may be reduced to two schemes. The first is that which is general in plants, and which Schwann believed to be as common in the animal tissues. In it there are first produced in the cytoblastema minute granules, perhaps simple cells. Around each one or more of these, an imperfect layer of substance gradually collects by a sort of coagulation of part of the cytoblastema, and this becomes gradually more dense till it forms a fine membrane around the primary granule. The membrane continues to grow, and increases more in superficial extent than in thickness; so that, after a time, a space is left between its inner surface and the granule. The space is usually filled with fluid, and the granule remains attached to some part of the membrane. In the next place, a second membrane forms upon the first, and, enlarging at a much greater rate than the first does, separates from it, appearing at first 'like a watch-glass upon a watch,' and then gradually rising and leaving a cavity which receives a fluid within it. Thus there is produced a membranous cell, inclosing or bearing on its wall a second much smaller cell, which again incloses, or bears on its wall, one or more granules or cells. The largest and last-formed is the *primary cell:* the next the *nucleus:* the third and smallest the *nucleolus.*

" The other scheme of development, which is probably the most frequent in the animal tissues, differs chiefly in the mode of formation of the nucleus. This is not developed as a cell upon a granule, but, of the granules which are first formed in the cytoblastema, a few (rarely more than three) collect, adhere together, and compose a nucleus, in which one granule larger than the rest seems sometimes to compose a nucleolus. On a nucleus thus formed, the cell develops itself in the manner already described; and, as the cell is developed, the granular character of the nucleus diminishes. The granules, which at first adhered very loosely, become inseparably united; and then, as if by the collection of all their solid material to their common circumference, they are gradually changed to a minute smoothly-walled vesicle filled with fluid.

"A scheme nearly analogous to this is observed in the globules of the first milk, and in some globules of pus and of inflammatory exudations, and several morbid products; in which a great number of granules collect together, and tend, or attain to the formation of a cell without any distinct nucleus. As in the preceding, the nucleus was formed without a distinct or previous nucleolus, so here, the development proceeds immediately by the aggregation of many similar granules to the formation of the cell.

"In whichever of these methods they be produced, the primary cells seem to possess the power of working upon their contents, upon each other, and upon the intercellular substance, and of effecting those diverse chemical and structural changes by which the infinite variety of organised tissues is produced. According to these changes Schwann divides all the elementary forms of animal structures into five chief divisions, as follows:—

"In the first, the form of the primary cells is nearly retained, and they either float separately in a fluid, or are moveable on each other. Of this division are the blood-, lymph-, mucus-, and pus-corpuscles, and the endogenous cells of certain glands, which, in their completest state, are primary cells floating in a fluid, and in which the changes after the formation of the cell are limited to those of their size and form, and those of their contents, in which, colouring and other matters, or new cells, are generated.

"In the second division, the primary cells are distinct, but they cohere by their walls, and sometimes firmly enough to form a connected tissue, as in the several varieties of cuticle, in pigment-membranes, and in fat. The changes of form which some of the cells in this class of tissues undergo, though greater than those of the preceding class, are simple. They assume a flattened, or an elongated, pyramidal, or conical form; they throw out processes which become ciliæ, or which, in some cases perhaps, as in certain pigment-cells, form canals of communication between the adjacent cells; or they are merely changed in form by their mutual pressure, which makes them angular, as in the epidermis-cells. Their contents also may be changed—becoming transparent, or having granules formed in them, or disappearing and permitting the cell to dry.

"In the first class, the fluid in which the cells float may be regarded as a permanent cytoblastema: in the second, the cytoblastema seems to be entirely or nearly consumed in the formation of cells; in some only of the tissues, a small quantity of it remains as a structureless intercellular substance, holding the cells together. In the third class, it becomes an important constituent of the tissue. The cells are nearly persistent in their primary form, but are separated from each other by the cytoblastema, which constitutes the chief mass of the tissue, and with which the walls of the cells are ultimately fused. Of this class are the bones and cartilages, of which the peculiar corpuscles are the primary cells, and the basis or intercellular substance in which they lie is the former cytoblastema. The chief differences in the cartilages depend on the relative quantity of the intercellular substance, and on its acquiring in its later development a finely fibrous structure. The canals of the bone-corpuscles either grow out from the sides of the cells, or are produced by spaces (intercellular passages) being left in the material deposited within the cells in the later period of their development.

"The fourth class is composed of tissues which in their adult state consist of fibres; the cells in which they originated having undergone an entire change in their appearance. In these, among which the chief examples are the varieties of fibro-cellular tissue, each primary cell becomes elongated and spindle-shaped, and its extremities are drawn out into fibres. These fibres sometimes branch, and, at the last, they break up by splitting from the end towards the centre where the several fissures meet, and form a bundle of fine filaments or tubules. In this way it is believed that from each primary cell a fasciculus of the filaments of fibro-cellular tissue is produced.

"The fifth and last class in Schwann's arrangement comprises the tissues which are formed from cells whose cavities, as well as their walls, have become connected together. In some of these the cells arrange themselves in rows, each being fixed to the ends of the two next to it; and the partitions which are at first formed by their apposed extremities being absorbed, the cavities of all the cells gradually merge into one. Thus, in the place of a row of primary cells, there is formed a single long cell, a secondary cell, whose wall is composed of the side walls of a series of primary cells. Such are the nerve-fibre, the muscular fibre, and the tubules of glands which have simple membranous walls. The secondary cell may grow like a primary cell, and may form various substances in its interior; the peculiar nerve substance, and the muscular fibrils, are supposed by Schwann to be deposited within the cavities of such secondary cells; and the seminal corpuscles, and the corpuscles of several other glands, may be placed in the same list of secondary intra-cellular formations.

"In another case belonging to this division, the cells, instead of arranging themselves in rows and coalescing end to end, lie scattered at some distance from each other, and, sending out processes from their sides into the surrounding cytoblastema, assume the shape of radiating cavities. Their processes coalescing, they become a network of canals, in which the differences of caliber, which at first existed, are gradually annulled. In this way, it is believed, the capillary blood-vessels are formed."

Such was Schwann's system. For the exceptions taken to it, as they do not materially affect it, we must refer to the Report itself. The views comprised in it touch very seriously our notions of the nutrition of the tissues. That would not seem to consist in the *action* of the blood-vessels. As Mr. Paget observes:—

"Tissues are not laid down as such, but each forms itself out of a similar structureless material, and none attains its perfect form without passing through several of the stages of development just described, over which blood-vessels seem to have no other influence than that of being, in certain cases, the conveyers of material. In the embryo, before blood exists or has been sent to any part, each part receives or consists of a similar material, which, by a power implanted in it according to a plan specially ordained for each creature, works itself into the peculiar form of each part. And when once a tissue is fully developed, the act of its nutrition is still the same. Each tissue abstracts from the blood, or otherwise receives, a similar material in the quantity necessary for its repair or growth, and this material, as in the embryonic state, develops itself. Only now its tendency to assume this or that form is influenced by the tissue adjacent to it; for, with its perfect development, each tissue seems to acquire a peculiar power of assimilation, that is, of determining another adjacent substance capable of development to assume the same form and composition which itself has.

"In the degeneration of tissues also, or in that which may be called their involution, as the contrary of their evolution or development, and which is preparatory to their removal from the body, they cannot be considered as *taken* up or absorbed, without change, by absorbent or other vessels. They probably retrograde through part of the stages through which they passed in their development, return to their former structureless or fluid state, and then mix with the fluid which is passing into the circulation for excretion, or for the nutriment of other parts."

### RECENT PLATES OF ANATOMY.

I. ATLAS ILLUSTRATIVE OF THE ANATOMY OF THE HUMAN BODY. By *J. Cruveilhier,* M.D. Professor of Anatomy to the Faculty of Medicine, Paris. The Figures drawn from Nature, by *Em. Beau.* With Explanations by *C. Bonamy,* M.D. Price 2s. 6d. plain, and 5s. coloured, each Part.

THIS Series of Plates has now attained some age and some dimensions, and the Subscribers have a guarantee for its continuation and completion. Indeed, the character of M. Baillière, the publisher, from the first gave sufficient warranty for such an expectation. In no instance has that gentleman broken faith with the public, but religiously brought to a conclusion what be had commenced.

Of the Plates themselves we can speak in high terms. They are accurate, spirited, and clear. Perhaps they are rather small in size, but the execution of the lithography does away with any objection on the score of indistinctness, and a lower scale of price is thus obtained. As far as precision and artistical merit go, they are equal to the plates of Bourgery, and very much more reasonable. It is to be hoped that M. Bourgery's mode of publication will not be imitated in this instance, Any thing more irregular, tantalising, and disingenuous could not well be conceived.

M. Cruveilhier's Plates have been brought to the 15th Part, and already exhibit most of the Apparatus of Locomotion—the Muscles and the Bones. We cordially recommend them.

II. THE ANATOMY OF THE ARTERIES OF THE HUMAN BODY, WITH ITS APPLICATIONS TO PATHOLOGY AND OPERATIVE SURGERY; IN LITHOGRAPHIC DRAWINGS, WITH PRACTICAL COMMENTARIES. By *Richard Quain,* Professor of Anatomy in University College, and Surgeon to University College Hospital. The Delineations by *Joseph Maclise,* Esq. Surgeon. Price, each Part, Twelve Shillings.

ON more than one occasion we have spoken in the highest possible terms of this splendid work. It reflects the greatest credit upon Mr. Quain, as well as on Mr. Maclise, the artist, and is really a national production. Every Book Club, every Medical Library, every Medical Public Institution should possess and patronize it. And every wealthy member of our profession should not fail to subscribe to a work like this, one of a class not sufficiently seen in this country.

On a future occasion we intend to present a connected view of the practical portion of the letter-press. In the mean time, we trust that our recommendations will not have been altogether in vain.

